# Supplementary material for: Endogenous MicroRNA Competition as a Mechanism of shRNA-Induced Cardiotoxicity
Source: Mol Ther Nucleic Acids. 2019 Dec 18;19:572–80. doi: 10.1016/j.omtn.2019.12.007 (PMC6957822; doi:10.1016/j.omtn.2019.12.007)
Supplement: Document S1. Figures S1–S5 [file mmc1.pdf]

OMTN, Volume *19*

Supplemental Information

Endogenous MicroRNA Competition

as a Mechanism of shRNA-Induced Cardiotoxicity

Meredith M. Course, Kathryn Gudsnuk, Nitin Desai, Joel R. Chamberlain, and Paul N. Valdmanis

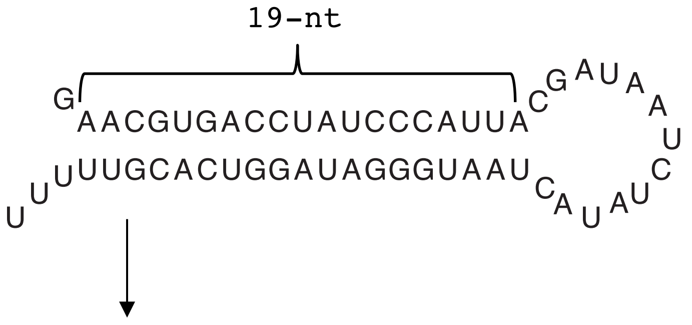

19-nt stem reads	Proportion of top reads
ATGGGATAGGTCACGTTTTTT	17.3%
ATGGGATAGGTCACGTTTTTTT	31.6%
AATGGGATAGGTCACGTTTTT	32.1%
AATGGGATAGGTCACGTTTTTT	14.4%

19-nt: GAACGTGACCTATCCCATTACG––ATAATCTATACT––AATGGGATAGGTCACGTTTTTT

21-nt: GAACGTGACCTATCCCATTACGTGATAATCTATACTGTAATGGGATAGGTCACGTTTTTT

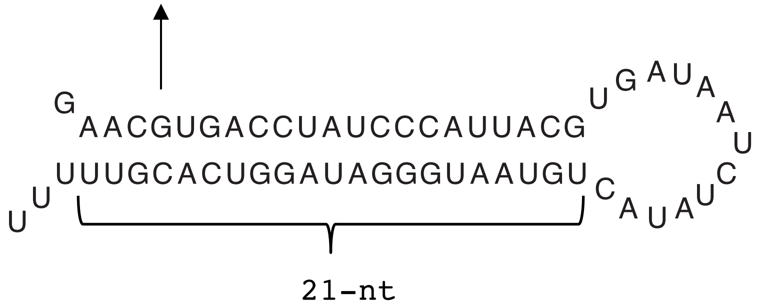

21-nt stem reads	Proportion of top reads
TAATGGGATAGGTCACGTTT	4.8%
TAATGGGATAGGTCACGTTTT	49.1%
TAATGGGATAGGTCACGTTTTT	33.7%
TAATGGGATAGGTCACGTTTTTT	7.6%

Figure S1. 19-nt and 21-nt sh-*lacZ* processing.
Shown are the predicted structures of the 19-nt and 21-nt hairpins, their flattened sequences, and the top four reads that are produced from the hairpins along with their relative abundance (n = 3, proportion is the mean average of reads in three hearts analyzed at 6-weeks post-injection). The reads produced from the 19-nt hairpin are not observed to an appreciable degree in the 21-nt-treated hearts, nor are the reads produced from the 21-nt hairpin observed in the 19-nt-treated hearts.

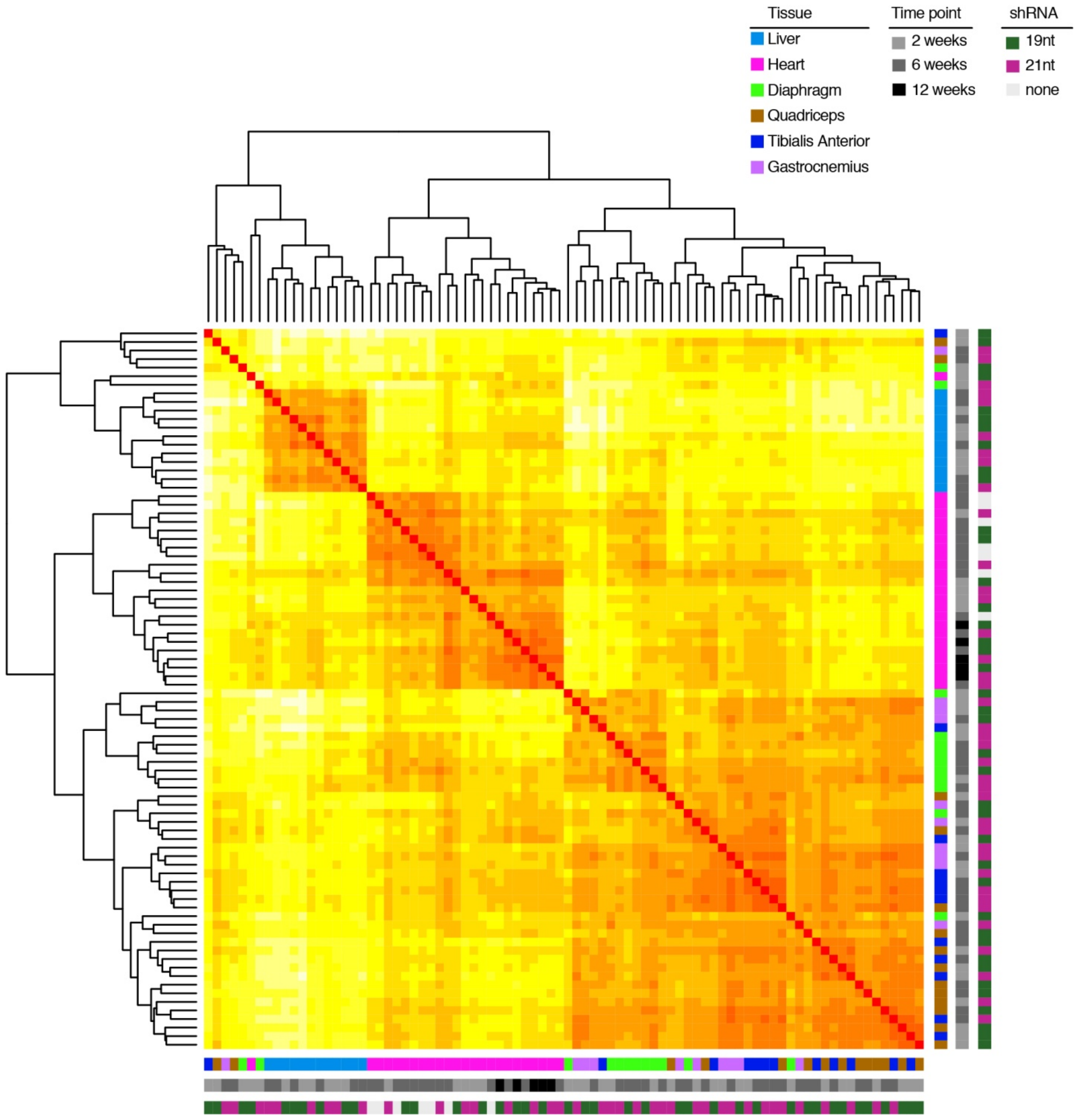

Figure S2. Hierarchical clustering of all samples. Tissue groups cluster into three broad categories: heart, liver, and all other muscles (diaphragm, quadriceps, tibialis anterior, and gastrocnemius). They do not cluster by time point (2, 6, or 12 weeks) or by treatment paradigm (no shRNA, 19-nt, or 21-nt).

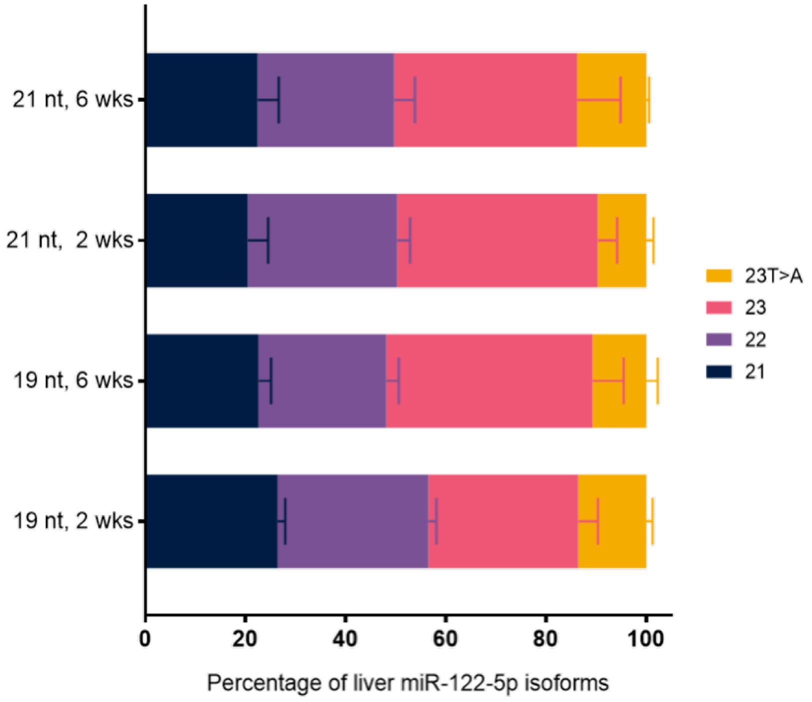

Figure S3. MiR-122-5p isoforms in liver tissues of 19-nt and 21-nt injected mice.
MiR-122-5p isoform expression in liver tissues after injection of the 19-nt shRNA and the 21-nt shRNA. The 22-nt isoform is not depleted relative to the 21-nt isoform. n = 3 mice per group. Data are mean ± SD.

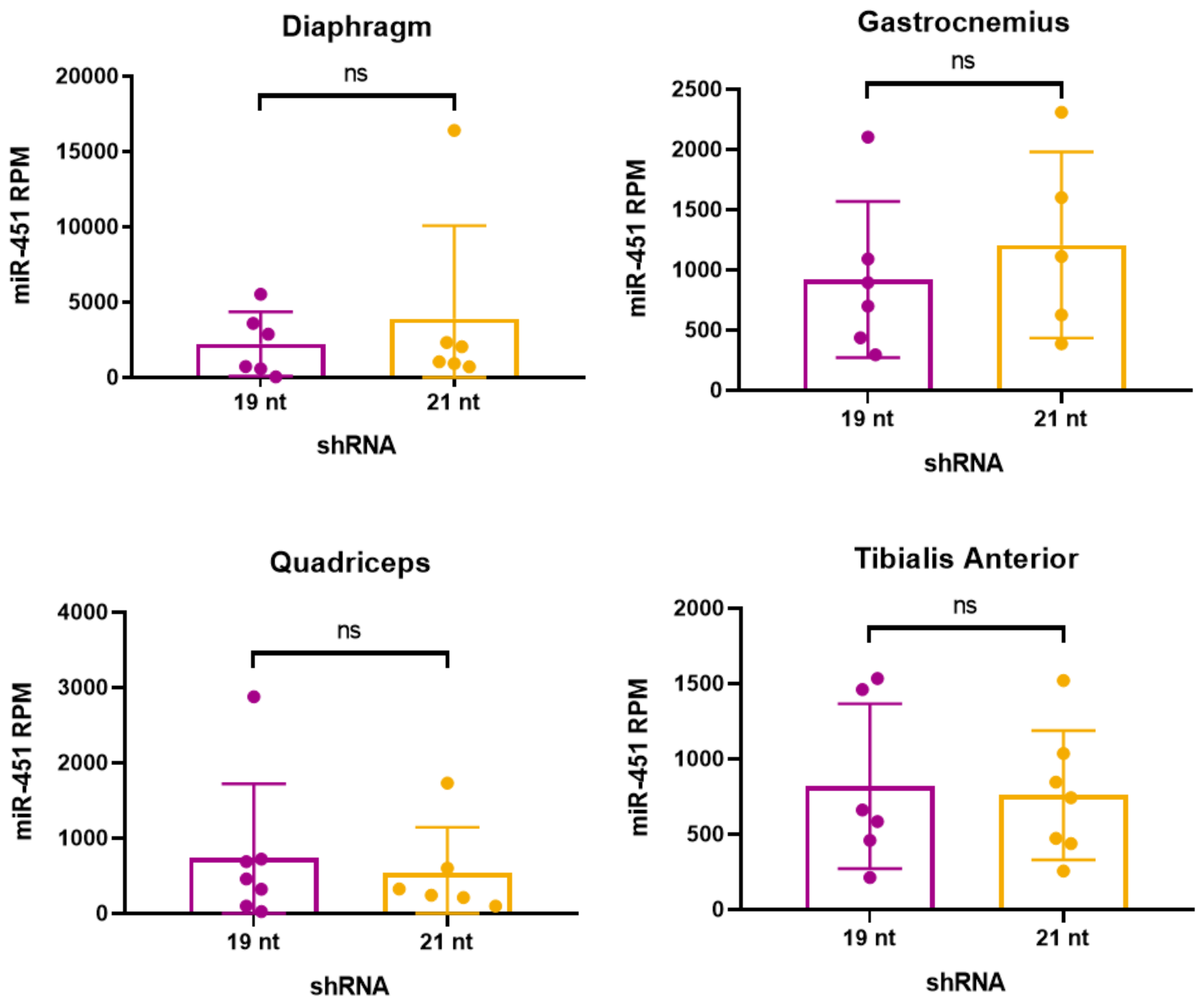

Figure S4. MiR-451 in muscle tissues of 19-nt and 21-nt injected mice.
MiR-451 expression in muscle tissues other than the heart after injection of the 19-nt shRNA and the 21-nt shRNA. n = 5-7 mice per group. Data are mean ± SD. RPM is reads per million. NS means "not significant," as determined by a Student's t-test.

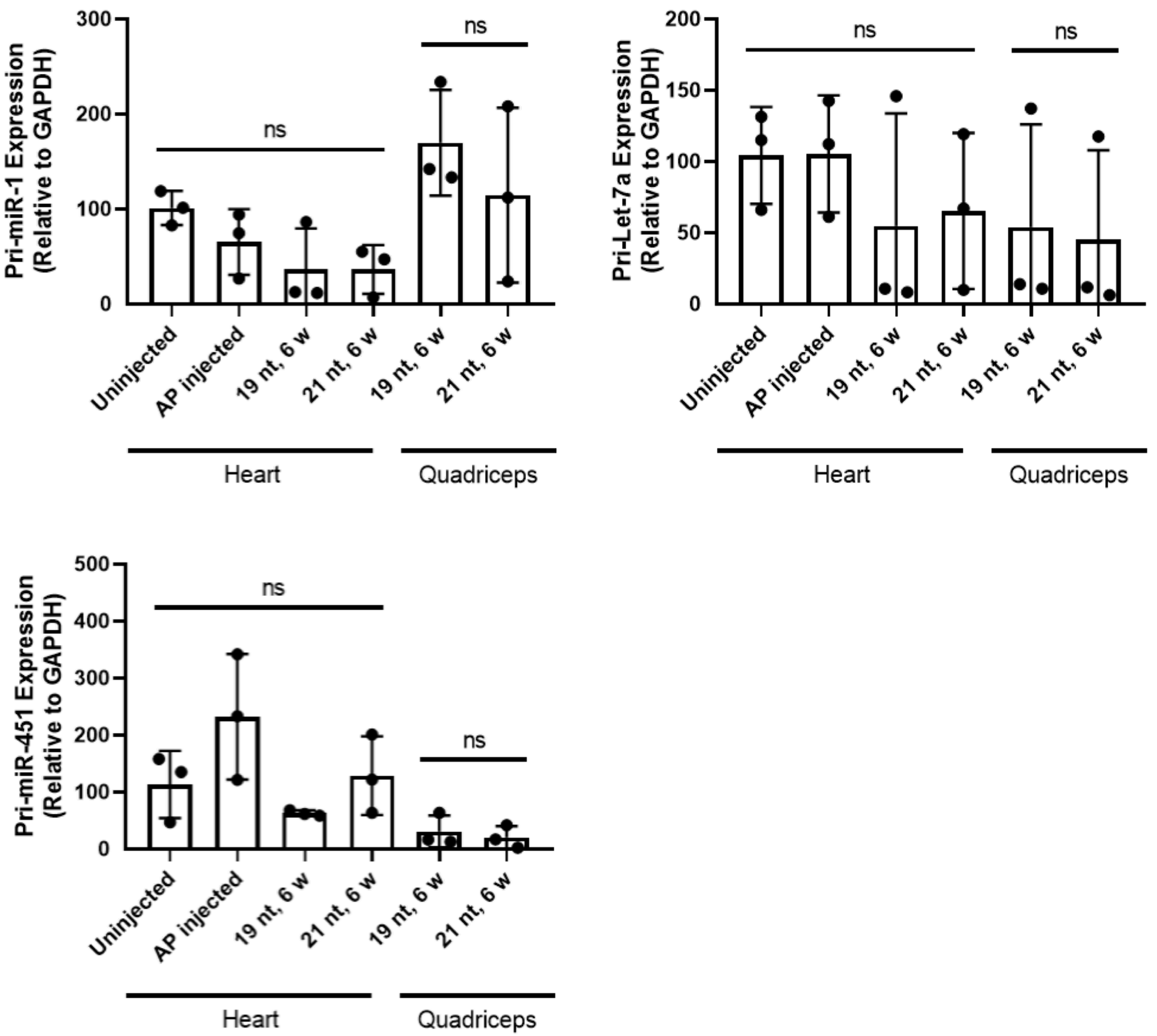

Figure S5. Primary miRNA transcripts in muscle tissues of 19-nt and 21-nt injected mice. qRT-PCR for primary miRNA transcripts in heart and quadriceps tissues of 19- and 21-nt injected mice. n = 3 mice per group. NS means "not significant." Analyses performed were one-way ANOVA tests followed by Tukey's multiple comparisons for heart tissue, and Student's t-tests for quadriceps tissue. Data are mean ± SD.
