# Supplementary material for: Endogenous MicroRNA Competition as a Mechanism of shRNA-Induced Cardiotoxicity
Source: Mol Ther Nucleic Acids. 2019 Dec 18;19:572–80. doi: 10.1016/j.omtn.2019.12.007 (PMC6957822; doi:10.1016/j.omtn.2019.12.007)
Supplement: Document S2. Article plus Supplemental Information [file mmc3.pdf]

Molecular Therapy
Nucleic Acids
Original Article

Endogenous MicroRNA Competition as a Mechanism of shRNA-Induced Cardiotoxicity

Meredith M. Course,[1] Kathryn Gudsnuk,[1] Nitin Desai,[1] Joel R. Chamberlain,[1,2] and Paul N. Valdmanis[1,2]

[1]Division of Medical Genetics, University of Washington School of Medicine, Seattle, WA, USA

Gene knockdown using short hairpin RNAs (shRNAs) is a promising strategy for targeting dominant mutations; however, delivering too much shRNA can disrupt the processing of endogenous microRNAs (miRNAs) and lead to toxicity. Here, we sought to understand the effect that excessive shRNAs have on muscle miRNAs by treating mice with recombinant adeno-associated viral vectors (rAAVs) that produce shRNAs with 19-nt or 21-nt stem sequences. Small RNA sequencing of their muscle and liver tissues revealed that shRNA expression was highest in the heart, where mice experienced substantial cardiomyopathy when shRNAs accumulated to 51.2% ± 13.7% of total small RNAs. With the same treatment, shRNAs in other muscle tissues reached only 12.1% ± 5.0% of total small RNAs. Regardless of treatment, the predominant heart miRNAs remained relatively stable across samples. Instead, the lower-expressed miR-451, one of the few miRNAs processed independently of Dicer, changed in relation to shRNA level and toxicity. Our data suggest that a protective mechanism exists in cardiac tissue for maintaining the levels of most miRNAs in response to shRNA delivery, in contrast with what has been shown in the liver. Quantifying miRNA profiles after excessive shRNA delivery illuminates the host response to rAAV-shRNA, allowing for safer and more robust therapeutic gene knockdown.

INTRODUCTION

DNA vectors expressing short hairpin RNAs (shRNAs) can be engineered and delivered to knock down the expression of dominant mutations through RNA interference (RNAi)-mediated cleavage of cognate mRNAs. During this process, shRNAs co-opt the cells' endogenous microRNA (miRNA) machinery, meaning that delivery of excessive shRNAs could monopolize the machinery and compete with the processing of endogenous miRNAs. Disruption of the endogenous miRNA levels, in turn, has been shown to lead to toxicity in several tissues, including the liver and the brain.[1–4] Therefore, the goal of RNAi therapies is to deliver enough shRNA and in such a manner that the treatment is effective, yet does not outcompete the miRNAs in the targeted tissue.

One of the first reported systemic deliveries of recombinant adeno-associated viral (rAAV)-shRNA to muscle was in an early model of facioscapulohumeral dystrophy (FSHD), where 40%–60% target mRNA knockdown in skeletal muscles led to a functional improvement back to wild-type levels.[5] Since then, several groups have delivered shRNAs or miRNA precursors in an attempt to knock down genes in the muscle. Some examples are the *DUX4* gene involved in FSHD,[6] the nuclear factor κB (NF-κB) gene in the mdx mouse model of Duchenne muscular dystrophy,[7] the RNA polymerase of the coxsackievirus B3 to prevent CoxB3-mediated cardiomyopathy,[8] the NADPH oxidase gene to prevent cold-induced hypertension in rats,[9] and the phospholamban (*PLB*) gene for improved calcium cycling to prevent heart failure.[10] Unfortunately, in this last example, cardiomyopathy was observed in canines receiving high levels of shRNA delivery against *PLB*,[10] and similar organ toxicity has been observed in other cases of shRNA delivery. Therefore, better understanding the mechanism behind shRNA-related toxicity, including the relative biodistribution of shRNAs in various muscle tissues and their effects on the endogenous miRNA environment, is an unresolved issue that needs to be addressed.

Here, we seek to understand how shRNA expression in muscle tissues affects the endogenous miRNA environment. We previously delivered rAAV vectors expressing shRNAs targeting the liver in mice, and discovered that liver toxicity was caused by shRNA competition with miR-122-5p miRNAs.[11] Although shRNA-induced toxicity occurs in tissues other than the liver, none of these tissues express miR-122 in notable levels, suggesting that toxicity is caused by an alternative mechanism. Our data indeed suggest that exogenous shRNAs compete with muscle miRNAs in a different manner than they do in the liver, leading to the observed cardiomyopathy.

RESULTS

To investigate the effects of exogenous shRNAs on endogenous muscle miRNAs, we assessed tissues from ROSA26 mice (which constitutively express the *lacZ* gene in all tissues) that had been injected via tail vein with 2×10^{12} vector genomes of rAAV6 expressing shRNAs, and that were described and characterized previously.[12] rAAV6 was used because it robustly transduces muscle tissues.[13] The shRNAs were driven by the U6 promoter

Received 17 September 2019; accepted 12 December 2019;
https://doi.org/10.1016/j.omtn.2019.12.007.

[2]These authors contributed equally to this work.

Correspondence: Paul N. Valdmanis, Division of Medical Genetics, University of Washington School of Medicine, 1705 NE Pacific Street, HSB J-305, Box 357720, Seattle, WA 98195-7720, USA.
E-mail: paulnv@uw.edu

 © 2019 The Author(s).
This is an open access article under the CC BY license (http://creativecommons.org/licenses/by/4.0/).

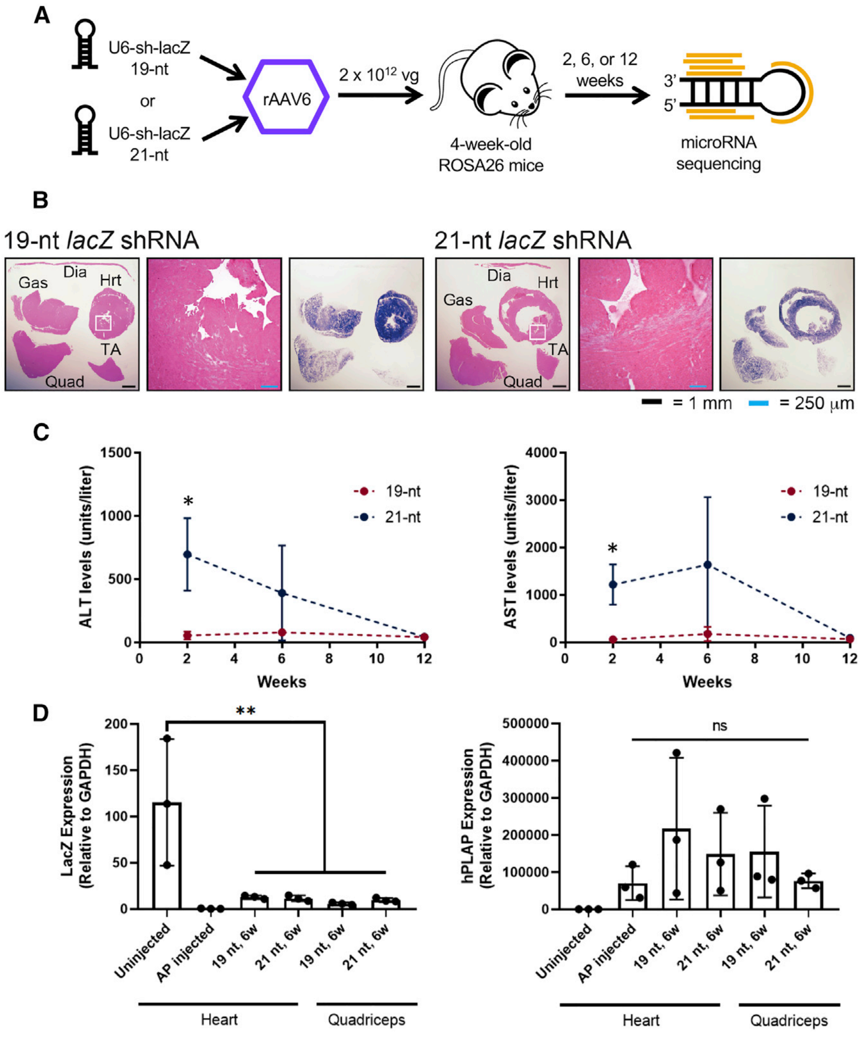

Figure 1. Twenty-one-Nucleotide shRNA Directed to Muscles Can Cause Toxicity in Mice

(A) Schematic of experimental design. (B) Histological muscle sections from 19-and 21-nt injected mice at 6 weeks post-injection. Left panels are H&E-stained sections, and the right panels are stained with human placental alkaline phosphatase (hPLAP). Sections shown are quadriceps (Quad), gastrocnemius (Gas), diaphragm (Dia), heart (Hrt), and tibialis anterior (TA). (C) Serum ALT and AST levels in 19-and 21-nt injected mice. ALT and AST levels are significantly higher in 21-nt injected mice as compared with 19-nt injected mice at 2 weeks post-injection (Welch's t test, p = 0.0201 and p = 0.0122, respectively), and resolve by 12 weeks post-injection. n = 3–4 mice per group. (D) qRT-PCR for lacZ and hPLAP levels in heart and quadriceps tissues of 19- and 21-nt injected mice at 6 weeks post-injection. LacZ expression is significantly reduced in tissues of treated animals to 5%–11% of untreated levels, and hPLAP levels are not significantly different. n = 3 mice per group. One-way ANOVA followed by Tukey's multiple comparisons. Data are mean ± SD.

and targeted *lacZ* mRNA, with either 19- or 21-nt complementary sequences. The vector also expressed a human placental alkaline phosphatase (*hPLAP*) reporter gene under the Rous sarcoma virus (RSV) promoter so transduction could be monitored. We also made use of existing tissues from HSA^{LR} mice injected only with *hPLAP* under the RSV promoter as one type of control, because the HSA^{LR} transgene is not expressed in the heart; these are referred to as "alkaline phosphatase (AP)-injected" samples, which were available only for heart tissue. We then performed small RNA sequencing on liver and muscle samples from mice at 2 and 6 weeks after shRNA administration (Figures 1A and S1). By 6 weeks, mice injected with the 19-nt shRNA vector showed minor amounts of mononuclear cells and mild focal necrosis, whereas those injected with the 21-nt shRNA exhibited substantial dilated cardiomyopathy with regional necrosis (Figure 1B). shRNA continued to accumulate in all muscle tissues over the 6-week period assessed (Figure 1B; described below), and two mice injected with the 21-nt shRNA died by 4 weeks post-injection and one by 8 weeks.[12] The 21-nt injection also led to transient toxicity in the liver, indicated by significantly increased alanine aminotransferase (ALT) and aspartate aminotransferase (AST) levels at 2 weeks (p = 0.0201 and p = 0.0122, respectively, Welch's t test), but this toxicity was resolved by 12 weeks after the 21-nt shRNA was eliminated (Figure 1C). At 6 weeks post-injection, *lacZ* expression was successfully reduced in quadriceps and heart tissues of animals treated with the 19-nt and 21-nt shRNA to 5%–11% of untreated levels, whereas *hPLAP* levels were not significantly different across these same tissues, confirming that transduction efficiency was similar (Figure 1D).

Small RNA sequencing of tissues at 2 and 6 weeks post-injection revealed that there was no significant difference in individual miRNA expression between mice treated with the 19-nt shRNA as compared with those treated with the 21-nt shRNA (reads were normalized to the combined shRNA and miRNA reads; Figure 2; Table S1). Hierarchical clustering of miRNAs revealed that liver samples cluster together, as do heart tissues. A third cluster is defined by all other muscle tissues, with some overlap between quadriceps, tibialis anterior, gastrocnemius, and diaphragm tissues. Samples did not cluster by time point or by 19-nt or 21-nt shRNA sequence, indicating that miRNAs broadly did not change (Figure S2). We therefore assessed overall miRNA changes in relation to the amount of shRNA in each tissue (Figure 3). Sequencing also allowed us to identify the major products of the 19-nt and 21-nt shRNAs (Figure S1). The predominant miRNA in the liver was

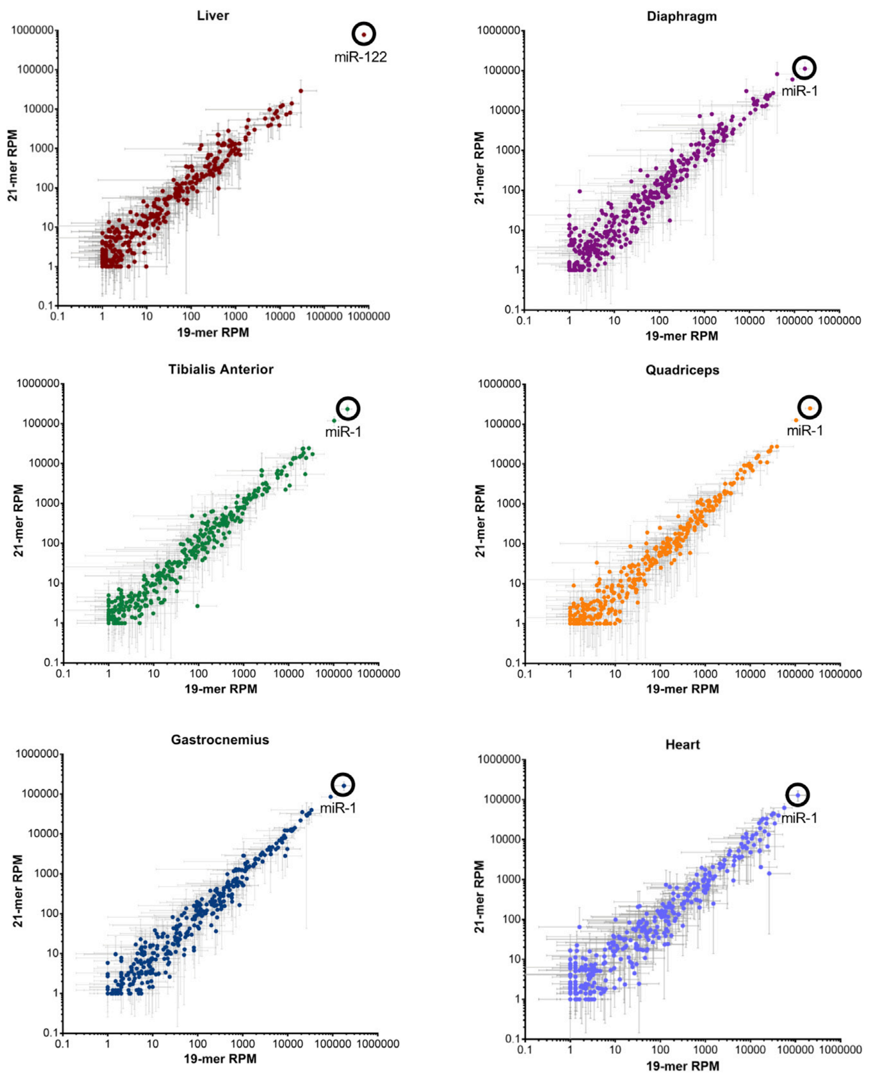

Figure 2. MicroRNA Expression in Muscle and Liver Tissues of shRNA-Treated Mice at 2 and 6 Weeks after Injection

Mean microRNA expression in tissues after injection of the 19-nt shRNA versus the 21-nt shRNA. n = 3 mice per group. Data are mean ± SD. In each graph, the predominant microRNA has been labeled. RPM, reads per million.

miR-122, which accounted for about 79.3% ± 5.9% of total miRNAs in the absence of shRNAs and decreased in direct proportion to shRNA increase, confirming previous findings (Figures 2 and 3A).[11] miR-122 isoforms remained unchanged, further indicating that the toxicity experienced by the mice was not due to disruption of liver miRNAs (Figure S3). The predominant miRNA in all muscle tissues was miR-1, which accounted for 12.7%–47.2% of all small RNAs across muscle types and treatments (Figures 2 and 3A). The predominant isoform of miR-1-3p in muscle tissues, in turn, was the 22-nt form, which accounted for about 73.1% ± 5.6% of all miR-1 across tissues (Figures 3B and 3C). miR-1 levels did not, however, alter in relation to the accumulated shRNA levels.

Heart tissues from animals treated with the 21-nt shRNA and assayed at 6 weeks (tissues that exhibited substantial dilated cardiomyopathy) contained levels of shRNAs accounting for about 51.2% ± 13.7% of total heart small RNAs. For this same treatment and time point, shRNAs in other muscle tissues were only 12.1% ± 5.0% of total small RNAs (Figure 3A). Due to the striking accumulation of shRNAs in the heart, and cardiomyopathy as the primary toxic event in these mice, we focused on identifying the mechanism of toxicity specifically in the heart tissue. For this tissue, we increased the experimental group to include samples taken at 12 weeks after shRNA administration.

Because levels of the top expressing miRNA did not change significantly with respect to shRNA accumulation in muscle tissues, we next examined the top four expressing miRNAs, to see whether any one of them changed individually, or if they changed as a group. The top four expressing miRNAs were miR-1, let-7, miR-133, and miR-378 (Figure 4A). Together, these four miRNAs accounted for 49.4% ± 9.4% of miRNAs in uninjected samples and samples injected with rAAV6-hPLAP; however, even as a group, these top miRNAs did not decrease in proportion to the increasing levels of shRNA accumulation. We confirmed this pattern by small RNA northern blotting, which demonstrated that absolute levels of these miRNAs were largely unchanged (Figure 4B). We therefore searched for any lower-expressing miRNA(s) that decreased in response to shRNA treatment.

To identify what miRNA this could be, we compared all miRNAs expressed in the hearts of the control mice versus the 21-nt injected mice. We found that miR-451 was significantly lower in 21-nt injected mice ($p = 0.0083$, one-way Kruskal-Wallis test; Figures 5A and 5B). miR-451 levels also trended down in the hearts of 19-nt injected mice, which had accumulated intermediate levels of shRNAs and exhibited mild regional necrosis, indicating that they reached a lower but non-toxic amount in these mice (Figure 5B). This trend was specific to the heart: miR-451 levels did not change in other muscle tissues (Figure S4).

To explore whether this decrease in miR-451 would affect expression of its targets, we probed for two confirmed protein

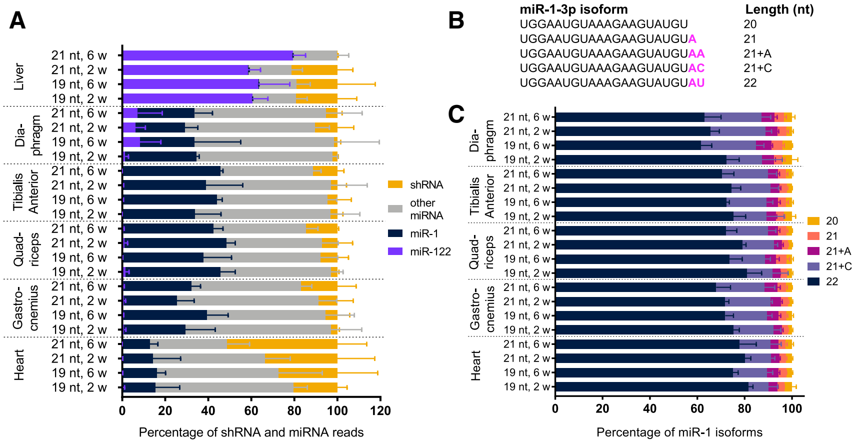

Figure 3. Small RNA Distribution in shRNA-Treated Mice

(A) Percentage of reads that map to shRNAs, miR-1, miR-122, and all other microRNAs. n = 3 mice per group. Data are mean ± SD. (B) Isoforms generated by miR-1-3p. (C) Percentage of miR-1-3p isoforms. n = 3 mice per group. Data are mean ± SD.

targets of miR-451, Cab39[14] and MIF,[15] in the heart tissues of uninjected mice and mice injected with the 19-nt and the 21-nt shRNA vectors at 6 weeks post-injection. As a control, we probed for two confirmed protein targets of miR-1: Cdk9[16] and Hand2[17] (Figures 5C and 5D). miR-1 target protein levels remained unchanged as expected, because miR-1 levels did not appreciably change across treatments. miR-451 target protein levels also remained unchanged, which is unsurprising given that it accounts for only 2.30% ± 2.36% of total shRNAs in the assayed tissues.

Finally, to determine whether the pattern of miRNA changes we observed could be related to changes in miRNA machinery, we evaluated the expression levels of *Drosha*, *Dicer1*, and *Argonaute 2* (*Ago2*; Figure 6). We also tested expression levels of primary miRNA transcripts for miR-1, Let-7a, and miR-451 (Figure S5). *Ago2* expression was significantly reduced in the tissues of treated animals as compared with their uninjected controls (p = 0.0022; one-way ANOVA followed by Tukey's multiple comparisons), but was not significantly different between 19-nt or 21-nt treatments in either heart or quadriceps. *Dicer1* levels were similarly lower in the tissues of treated animals versus uninjected controls, but were not significantly different among any groups. *Drosha* levels were not significantly different across groups. Primary miRNA transcripts matched levels of mature miRNAs in the heart and quadriceps with the exception of miR-451, which had similar levels of primary transcript expression despite reduced mature miRNA levels (Figure S5).

DISCUSSION

In this study, we sought to understand the effects of excessive shRNAs on muscle miRNAs, using a small RNA sequencing strategy that captured both exogenous shRNAs and endogenous miRNAs (Figure 1A). We found that cardiomyopathy arose when shRNAs exceeded 51.2% ± 13.7% of total heart miRNAs, at which level shRNAs in other muscle tissues reached only 12.1% ± 5.0% of total small RNAs and exhibited little degeneration (Figures 1B and 3A). Our observation of dilated cardiomyopathy is consistent with mouse studies of cardiac-specific *Dicer1* knockout[18] and of *Dgcr8* knockout, which impairs the *Dgcr8/Drosha* microprocessor complex.[19] The activation of an immune response and attempted clearance of the rAAV and sclerosis cannot be excluded as contributing factors to the cardiomyopathy, although so far studies have not shown that immune pathways are notably affected in response to AAV serotypes that transduce muscle tissue.[20] The comparable level of hPLAP across conditions also indicates that rAAV levels persist in hearts experiencing toxicity. miR-1 was the most abundant miRNA in the muscles

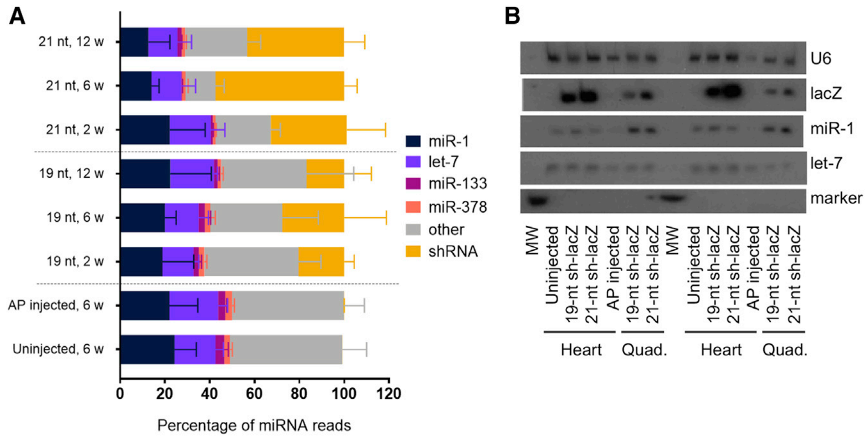

Figure 4. Small RNA Expression in Hearts of shRNA-Treated Mice

(A) MicroRNA levels in the hearts of 19-mer and 21-mer injected mice, as determined by deep sequencing. n = 3 mice per group, except for 21-nt, 12 weeks, for which n = 2 due to premature death of an animal. Data are mean ± SD. (B) Representative northern blots (of n = 3 biological replicates run three times) showing microRNA levels in the hearts of 19-mer and 21-mer injected mice in different biological replicates at 6 weeks post-injection. "MW" is a synthesized 21-nt RNA marker. AP, alkaline phosphatase.

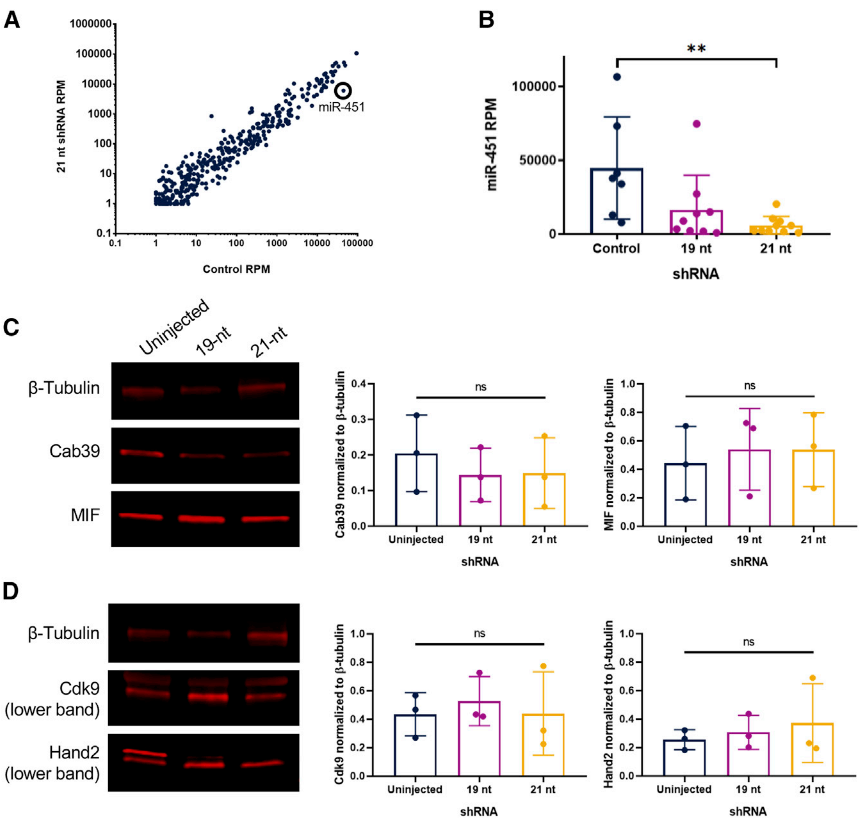

Figure 5. Protein Expression in Heart Tissues of shRNA-Treated Mice at 6 Weeks Post-injection

(A) Mean microRNA levels in the hearts of mice injected with the 21-mer shRNA as compared with controls. (B) miR-451 levels are significantly lower in the hearts of mice injected with the 21-mer shRNA as compared with controls (p = 0.0083, one-way Kruskal-Wallis test). n = 7–10 mice per group. Data are mean ± SD. (C and D) Representative western blots and quantification of western blots for control and shRNA-treated mouse heart tissues probed with (C) miR-451 targets Cab39 and MIF, and (D) miR-1 targets Cdk9 and Hand2. n = 3 blots. Data are mean ± SD. Significance was determined by a one-way ANOVA test. NS, not significant; RPM, reads per million mapped microRNAs.

of the injected mice; the most abundant isoform of miR-1-3p was 22 nt long and its levels remained constant regardless of treatment (Figure 3). miR-1 has previously been shown to be muscle specific and critical to heart function,[17,21–24] and here we show that in addition to being the most abundant miRNA in the heart, its levels remain the same regardless of shRNA level, indicating that there are factors in place to maintain its levels.

miR-1 abundance was closely followed by let-7, miR-133, and miR-378 (Figure 4). Previously, let-7 was shown to play a role in cardiac health,[25] and miR-133 was shown to play a role in skeletal muscle health.[21] When combined with miR-1, these four miRNAs comprise about half (49.4% ± 9.4%) of all heart miRNAs. The levels of these other three miRNAs similarly remain the same, regardless of shRNA level. In fact, it appears that the heart miRNAs decrease in response to shRNA level en masse, rather than one miRNA decreasing proportionally to the shRNA increase. We posit that there may be a protective mechanism for keeping the levels of these miRNAs stable because cardiac myocytes do not regenerate and therefore cannot recover from major changes in homeostasis.

miR-122 was the dominant miRNA in the liver, confirming what has been shown previously[11,23] (Figures 2 and 3A). In the event of hepatotoxicity mediated by AAV8-U6-shRNAs, the ratio of the most abundant 22-nt isoform of miR-122-5p is reduced compared with the 21-nt isoform. In this study, however, the miR-122-5p 22:21-nt isoform ratio is unchanged even at 2 weeks when ALT and AST levels are high, providing additional evidence that the muscle toxicity is the primary issue (Figure S3). Changes in miR-122 are responsible for hepatotoxicity in response to excessive shRNA treatment; however, hepatocytes experience rapid turnover, which could explain the difference in miRNA maintenance between tissues. Interestingly, the diaphragm expressed a notable amount of miR-122 as well. To confirm whether this had been seen elsewhere, we queried GEO: GSE36257,[26] which revealed that miR-122 was similarly within the 10 highest expressed miRNAs in the diaphragm.

Unlike toxicity in the liver mediated by AAV8-U6-shRNAs, the highest expressing miRNA was not responsible for toxicity in the heart. Instead, the lesser expressed miR-451 was reduced in response to increasing shRNA level (Figures 5A and 5B), although it is possible that miR-451 is simply deregulated and not crucial to toxicity. miR-451 levels were unchanged in other muscle tissues (Figure S4), likely because the heart is transduced more efficiently by rAAV6,[20] and thus experienced far more shRNA accumulation. Skeletal muscle also undergoes more regeneration than cardiac muscle. Although miR-451 was expressed in low levels in the heart tissue, we asked whether its decrease would affect the expression of confirmed protein targets Cab39[14] and MIF,[15] and found that the change in miR-451 levels did not significantly affect target protein levels (Figures 5C and 5D). miR-451 is unusual in that it does not require Dicer1 for processing and instead relies on the catalytic activity of Ago2.[27,28] The significant reduction in *Ago2* gene expression in both the 19-nt and 21-nt tissues (Figure 6) may therefore explain why we observe reduced miR-451 expression in these tissues. Ago2-mediated processing of miR-451 has prompted researchers to design an shRNA construct that can be processed in a similar manner,[29] and this study supports the need to further evaluate Dicer-independent machinery.

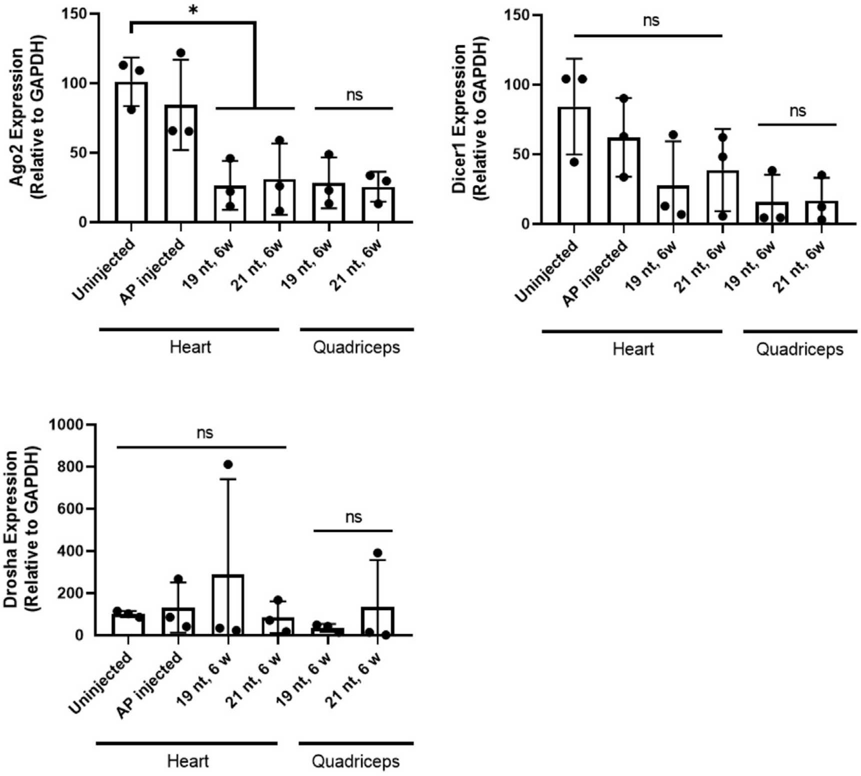

Figure 6. *Ago2*, *Dicer1*, and *Drosha* Levels Are Unchanged by 19-nt versus 21-nt Treatment

qRT-PCR for *Ago2* and *Dicer1* levels in heart and quadriceps tissues of 19-nt and 21-nt injected mice. *Ago2* expression is significantly reduced in tissues of treated animals versus uninjected controls (*p = 0.0022), but is not significantly different between 19-nt and 21-nt treatments in either heart or quadriceps. *Dicer1* levels similarly trend down in tissues of treated animals versus uninjected controls, but are not significantly different among any groups. n = 3 mice per group. Analyses performed were one-way ANOVA tests followed by Tukey's multiple comparisons for heart tissue and Student's t tests for quadriceps tissue. Data are mean ± SD. NS, not significant.

To take a closer look at the shRNA and miRNA processing machinery in these tissues, we analyzed the expression levels of *Drosha*, *Dicer1*, and *Ago2*. Even when shRNAs reached about 51.2% of total small RNA levels (in the heart at 6 weeks after injection with the 21-nt shRNA), *Drosha*, *Dicer1*, and *Ago2* levels were not significantly changed when compared with their levels in tissues with the 19-nt shRNA, which exhibits considerably less shRNA accumulation. This lack of change indicates that an upregulation of *Drosha*, *Dicer1*, or *Ago2* is not a compensatory response in the muscle to high levels of shRNAs (Figure 6). That said, it may still be worthwhile to try to upregulate one or both of these enzymes in the muscle to see whether they can facilitate tolerance of the shRNA treatment, as has been done in the liver.[30]

This study also provides a framework for the upper limits of shRNA-based expression (with 2×10^{12} vector genomes) that can be attained in skeletal muscles when providing a high dose of rAAV6 vector, a muscle-tropic vector currently being used in some gene therapy clinical trials. Its upper limits can be determined by treatment with the 19-nt sequence, which is minimally toxic in cardiac muscle, and can reach 4.93% ± 2.65% of total small RNAs in non-heart muscle tissue. shRNA levels in the non-heart tissues treated with the 21-nt shRNA sequence reach 12.1% ± 5.0% of total small RNAs, but are excessive in heart tissue with this delivery method (Figure 3A). If antagomirs or similar sequences could be expressed from a cardiac-specific promoter in the same vector, then perhaps higher dosing in skeletal muscles could be achieved while avoiding cardiotoxicity. Importantly, shRNA levels continued to accumulate in all striated muscle tissues during the period they were assessed, a long-term toxicity factor that should be considered when dosing cardiac tissue.

The 21-nt shRNA accumulated at high levels and led to cardiomyopathy, whereas the 19-nt shRNA accumulated at a lower level and was not toxic, likely because the 19-nt shRNA is a less efficient target for Dicer1. Therefore, this study reinforces the need for shRNAs to be designed either with a longer stem structure that first needs to be processed by Drosha, with a 19-nt stem that cannot be cleaved as efficiently by Dicer1, or with a less robust promoter, like the H1 RNA polymerase III promoter or RNA polymerase II promoter.[31] miRNA scaffolds are a recent option for circumventing shRNA-related toxicity;[32] however, the level of expression, and thus gene knockdown for miRNA scaffolds, is lower than it is for shRNAs. Notably, long-term expression of vector-delivered miRNAs is associated with cardiac arrhythmia and death in pigs, although whether the cardiac complications are a direct cause of the miRNA is unclear.[33]

Overall, rAAV6 transduces the heart at higher levels than other muscle tissues,[20] and is thus the key tissue to consider when designing shRNAs for muscle gene knockdown. In fact, most muscle-tropic AAV serotypes have much higher transduction of cardiomyocytes,[20] so their effects on these non-regenerating cells should be a primary concern, even when designing shRNAs to reduce gene expression in a different type of muscle. On the other hand, toxicity in the liver is resolved by the time the 21-nt injected mice die of cardiomyopathy, as indicated by ALT and AST levels (Figure 1C); therefore, the mechanism of transient AAV6-mediated liver toxicity is different from AAV8-mediated liver shRNA toxicity, which revolves around miR-122 levels. By quantifying muscle miRNA profiles after excessive shRNA delivery, we have illuminated the host response to muscle-targeting rAAV-shRNAs and identified a key challenge for muscle gene therapy: sufficiently transducing peripheral muscles without overloading cardiac muscle. Characterizing the limits of shRNA

Table 1. Antibodies Used for Western Blotting

Antibody	Company	Concentration Used
Anti-CAB39	Cell Signaling Technology, Danvers, MA, USA	1:750
Anti-MIF	R&D Systems, Minneapolis, MN, USA	1:750
Anti-Cdk9	Santa Cruz Biotechnology, Dallas, TX, USA	1:1,000
Anti-dHand	Santa Cruz Biotechnology, Dallas, TX, USA	1:500
Anti-beta Tubulin Loading Control	Life Technologies, Carlsbad, CA, USA	1:2,000
Goat anti-Mouse IgG (H+L) Highly Cross-Adsorbed Secondary Antibody, Alexa Fluor Plus 680	Life Technologies, Carlsbad, CA, USA	1:5,000
Goat anti-Rabbit IgG (H+L) Cross-Adsorbed Secondary Antibody, Alexa Fluor 680	Life Technologies, Carlsbad, CA, USA	1:5,000

accumulation in striated muscles and the miRNA response to that accumulation will help to overcome this challenge.

MATERIALS AND METHODS

shRNA Design and Generation

shRNAs vectors were designed, generated, and injected previously,[12] following the protocol detailed in Harper and Davidson.[34] In brief, the *lacZ* shRNA expression cassette was generated with DNA nucleotide extension of overlapping antisense oligonucleotides, followed by ligation into a plasmid containing the mouse U6 constitutive promoter. Vectors containing the 19-nt targeting sequence varied by a 2-nt truncation at the 5′ end of the siRNA guide strand. Each RNAi expression cassette was blunt-end ligated at a SnaBI restriction endonuclease site 5′ to the RSV-hPLAP reporter gene in the AAV plasmid pARAP4, which contains AAV serotype 2 inverted terminal repeats. The 19-nt and 21-nt *lacZ* sequences are shown in Figure S1.

Vector Design and Generation

Vectors were designed and generated previously.[12] Vectors were produced by shRNA plasmid and pDG6 (AAV6 capsid) plasmid cotransfection of subcultured HEK293 cells, as described in Blankinship et al.[13] In brief, rAAV6 shRNA vectors were harvested from cell pellets with homogenization followed by passing the homogenate through a 0.22-μm filter and further purification with HiTrap heparin column chromatography on an AKTA10 high-performance liquid chromatography (HPLC) machine (Amersham, Piscataway, NJ, USA). Vectors were titered by heating aliquots at 95°C for 10 min and subjected to agarose gel electrophoresis and Southern blotting analysis using the *hPLAP* SV40 polyadenylation sequence ^{32}P-labeled antisense DNA probe. Viral genomes were quantitated using phosphor-imager analysis (Storm 860; General Electric, Boston, MA, USA) relative to a DNA standard of the same molecular weight.

Vector Injections and Mouse Tissue Analysis

The Institutional Animal Care and Use Committee of the University of Washington approved all animal experimental procedures. Vectors were injected previously.[12] Purified shRNA vectors were administered systemically to ROSA26 and HSA^{LR} mice (Jackson Laboratories, Bar Harbor, ME, USA) via tail vein injection at 2×10^{12} vector genomes. HSA^{LR} mice express a human skeletal actin gene carrying a microsatellite expansion in the 3′ UTR as a model of myotonic dystrophy in skeletal muscle, but do not express the transgene in cardiac muscle. Mouse tissues were harvested at 2, 6, or 12 weeks post-injection and flash frozen in LN_2 for subsequent RNA isolation or frozen in optimal cutting temperature compound (OCT) for cryopreservation. For histological stain analyses, cryopreserved tissues were cut with a microtome into 10-μm sections and stained either with hematoxylin and eosin (H&E) or for human AP activity to estimate vector transduction levels *in vivo* as described previously.[5]

Table 2. Primers Used for RT-PCR and qRT-PCR

Target	Forward Primer	Reverse Primer
Ago2	5′-CTACAAGTCCACCCGCTTCA-3′	5′-TCATGGTGGAGAACCTGCTG-3′
Dicer1	5′-AAGTGGGCTGTATGAGAGATTG-3′	5′-GGCAGTCTGAGAGGATTTGTT-3′
Drosha	5′-CATGCACCAGATCCTTCTCTAC-3′	5′-TCGTGTTCTTCTGCCGTATTT-3′
hPLAP	5′-GGTGAACCGCAACTGGTACT-3′	5′-CCCACCTTGGCTGTAGTCAT-3′
lacZ	5′-GGCGTATCGCCAAAATCACC-3′	5′-ATGGGTAACAGTCTTGGCGG-3′
GAPDH	5′-TCAAGAAGGTGGTGAAGCAGG-3′	5′-ACCAGGAAATGAGCTTGACAAA-3′
pri-miR-1a	5′-CCTGTCTGCTTCCAGTCTTTAC-3′	5′-ATCGGTCCATTGCCTTTCC-3′
pri-let7a	5′-TGATGCTCAGCTGTGATTACTT-3′	5′-ACTGCAGTTTGTTCTTGGTTTC-3′
pri-miR-451a	5′-CACTGCTCGGCCTAATCAA-3′	5′-TTGGCACAGTGAAGAGGAAA-3′

ALT and AST Measurements

Mouse serum was collected by retro-orbital bleed (200 μL/mouse) in anesthetized mice. ALT and AST levels in samples were then assessed by Phoenix Central Laboratories (Mukilteo, WA, USA).

Small RNA Sequencing and Analysis

Muscle samples were dissected on dry ice and immediately homogenized in QIAzol Lysis Reagent (QIAGEN, Germantown, MD, USA) to extract RNA. Small RNA sequencing was performed as previously described.[11,35] In brief, 3 (liver) or 1 μg (muscle) of RNA was ligated to 3′ Universal miRNA Cloning Linker (New England Biosciences, Ipswich, MA, USA) using T4 RNA Ligase 1 (New England Biosciences, Ipswich, MA, USA) without ATP, then run on an 8M urea-15% polyacrylamide gel. Seventeen- to twenty-eight-nucleotide fragments were excised and ligated to 5′ barcodes again using T4 RNA ligase, then multiplexed and sequenced on an Illumina miSeq machine obtaining 50-bp single-end reads at the University of Washington Center for Precision Medicine. Linkers and barcodes were trimmed from the sequences; then the sequences were aligned to mouse miRNAs on miRBase (release 15)[36] using Bowtie version 0.12.7, allowing for two mismatches.[37] Hierarchical clustering of miRNAs was performed using the DESeq R package.[38] Small RNA sequencing data have been deposited in the NCBI Gene Expression Omnibus (GEO) repository with accession number GEO: GSE129896.

Western Blotting

Protein was extracted from mouse tissues using Tissue Protein Extraction Reagent (T-PER) plus Halt Protease Inhibitor Cocktail (both from Thermo Scientific, Waltham, MA, USA), following manufacturer's instructions. A total of 50 μg of protein per sample was run on 4%–20% Mini-PROTEAN TGX Gels and transferred using the Trans-Blot Turbo Transfer System and Nitrocellulose RTA Transfer Kit (all from Bio-Rad Laboratories, Hercules, CA, USA). Membranes were then immunoblotted with the antibodies listed in Table 1.

Blots were imaged on an Odyssey CLx Imager (LI-COR Biosciences, Lincoln, NE, USA). Quantification was performed using ImageJ 1.46r (NIH).

Northern Blotting

RNA was extracted from mouse tissues using QIAzol Lysis Reagent (QIAGEN, Germantown, MD, USA), size separated by 8M urea-15% polyacrylamide gel electrophoresis, and then wet transferred to Hybond-XL membranes (GE Healthcare, Chicago, IL, USA). miRNAs were detected using the following ^{32}P-labeled antisense probes: lacZ, 5′-GAACGTGACCTATCCCATTA-3′; let-7a-5p, 5′-GACTAT ACAACCTACTACCTC-3′; miR-1-3p, 5′-GTACATACTTCTTTAC ATTCC-3′; miR-122-5p, 5′-GAAACACCATTGTCACACTCC-3′; and U6, 5′-GTATATGTGCTGCCGAAGCGA-3′.

RT-PCR and qRT-PCR

For RT-PCR, RNA was extracted from mouse tissues using QIAzol Lysis Reagent (QIAGEN, Germantown, MD, USA), converted to cDNA using ProtoScript II First Strand cDNA Synthesis Kit (New England Biosciences, Ipswich, MA, USA), and PCR amplified using AllTaq Master Mix Kit (QIAGEN, Germantown, MD, USA). For qRT-PCR, the same cDNA was amplified using the Luna Universal qPCR Master Mix Kit (New England Biosciences) and run on a CFX384 Touch Real-Time PCR Detection System (Bio-Rad Laboratories, Hercules, CA, USA). Fold changes were calculated using the $\Delta\Delta C_T$ method.[39] Primers used for both assays are listed in Table 2.

Statistical Analyses

Statistical analyses were performed using Prism 8.0.1 (GraphPad Software, San Diego, CA, USA). Data in bar graphs are represented as mean ± standard deviation. To test whether the data were normally distributed, we used the D'Agostino-Pearson omnibus K2 test for $n \geq 8$, and the Shapiro-Wilk test was used for $n < 8$. A one-way ANOVA test was used to compare groups greater than two with parametric distribution, followed by Tukey's multiple comparisons test if the ANOVA gave $p < 0.05$. A one-way Kruskal-Wallis test was used to compare groups greater than two with nonparametric distribution, followed by Dunn's multiple comparisons test if the Kruskal-Wallis test gave $p < 0.05$. Student's t tests were used to compare unpaired groups of two with equal variances. Welch's t tests were used to compare unpaired groups of two with unequal variances.

SUPPLEMENTAL INFORMATION

Supplemental Information can be found online at https://doi.org/10.1016/j.omtn.2019.12.007.

AUTHOR CONTRIBUTIONS

M.M.C., J.R.C., and P.N.V. designed the experiments and analyzed the data. M.M.C., K.G., and N.D. performed the experiments. M.M.C. and P.N.V. wrote the manuscript with input from all co-authors.

CONFLICTS OF INTEREST

The authors declare no competing interests.

ACKNOWLEDGMENTS

We would like to thank all of the members of the Valdmanis lab for their assistance in the preparation of this manuscript. We would also like to thank Andre Lieber and the members of his lab for providing access to equipment and reagents.

REFERENCES

1. Ehlert, E.M., Eggers, R., Niclou, S.P., and Verhaagen, J. (2010). Cellular toxicity following application of adeno-associated viral vector-mediated RNA interference in the nervous system. BMC Neurosci. *11*, 20.

2. Grimm, D., Streetz, K.L., Jopling, C.L., Storm, T.A., Pandey, K., Davis, C.R., Marion, P., Salazar, F., and Kay, M.A. (2006). Fatality in mice due to oversaturation of cellular microRNA/short hairpin RNA pathways. Nature *441*, 537–541.

3. Borel, F., van Logtenstein, R., Koornneef, A., Maczuga, P., Ritsema, T., Petry, H., van Deventer, S.J.H., Jansen, P.L.M., and Konstantinova, P. (2011). In vivo knock-down of multidrug resistance transporters ABCC1 and ABCC2 by AAV-delivered shRNAs and by artificial miRNAs. J. RNAi Gene Silencing *7*, 434–442.

4. Martin, J.N., Wolken, N., Brown, T., Dauer, W.T., Ehrlich, M.E., and Gonzalez-Alegre, P. (2011). Lethal toxicity caused by expression of shRNA in the mouse striatum: implications for therapeutic design. Gene Ther. *18*, 666–673.

5. Bortolanza, S., Nonis, A., Sanvito, F., Maciotta, S., Sitia, G., Wei, J., Torrente, Y., Di Serio, C., Chamberlain, J.R., and Gabellini, D. (2011). AAV6-mediated systemic shRNA delivery reverses disease in a mouse model of facioscapulohumeral muscular dystrophy. Mol. Ther. *19*, 2055–2064.

6. Wallace, L.M., Liu, J., Domire, J.S., Garwick-Coppens, S.E., Guckes, S.M., Mendell, J.R., Flanigan, K.M., and Harper, S.Q. (2012). RNA interference inhibits DUX4-induced muscle toxicity in vivo: implications for a targeted FSHD therapy. Mol. Ther. *20*, 1417–1423.

7. Yang, Q., Tang, Y., Imbrogno, K., Lu, A., Proto, J.D., Chen, A., Guo, F., Fu, F.H., Huard, J., and Wang, B. (2012). AAV-based shRNA silencing of NF-κB ameliorates muscle pathologies in mdx mice. Gene Ther. *19*, 1196–1204.

8. Fechner, H., Sipo, I., Westermann, D., Pinkert, S., Wang, X., Suckau, L., Kurreck, J., Zeichhardt, H., Müller, O., Vetter, R., et al. (2008). Cardiac-targeted RNA interference mediated by an AAV9 vector improves cardiac function in coxsackievirus B3 cardiomyopathy. J. Mol. Med. (Berl.) *86*, 987–997.

9. Wang, X., Skelley, L., Wang, B., Mejia, A., Sapozhnikov, V., and Sun, Z. (2012). AAV-based RNAi silencing of NADPH oxidase gp91(phox) attenuates cold-induced cardiovascular dysfunction. Hum. Gene Ther. *23*, 1016–1026.

10. Bish, L.T., Sleeper, M.M., Reynolds, C., Gazzara, J., Withnall, E., Singletary, G.E., Buchlis, G., Hui, D., High, K.A., Gao, G., et al. (2011). Cardiac gene transfer of short hairpin RNA directed against phospholamban effectively knocks down gene expression but causes cellular toxicity in canines. Hum. Gene Ther. *22*, 969–977.

11. Valdmanis, P.N., Gu, S., Chu, K., Jin, L., Zhang, F., Munding, E.M., Zhang, Y., Huang, Y., Kutay, H., Ghoshal, K., et al. (2016). RNA interference-induced hepatotoxicity results from loss of the first synthesized isoform of microRNA-122 in mice. Nat. Med. *22*, 557–562.

12. Wei, J., and Chamberlain, J.R. (2014). Systemic RNAi delivery to the muscles of ROSA26 mice reduces lacZ expression. PLoS ONE *9*, e102053.

13. Blankinship, M.J., Gregorevic, P., Allen, J.M., Harper, S.Q., Harper, H., Halbert, C.L., Miller, A.D., and Chamberlain, J.S. (2004). Efficient transduction of skeletal muscle using vectors based on adeno-associated virus serotype 6. Mol. Ther. *10*, 671–678.

14. Godlewski, J., Nowicki, M.O., Bronisz, A., Nuovo, G., Palatini, J., De Lay, M., Van Brocklyn, J., Ostrowski, M.C., Chiocca, E.A., and Lawler, S.E. (2010). MicroRNA-451 regulates LKB1/AMPK signaling and allows adaptation to metabolic stress in glioma cells. Mol. Cell *37*, 620–632.

15. Bandres, E., Bitarte, N., Arias, F., Agorreta, J., Fortes, P., Agirre, X., Zarate, R., Diaz-Gonzalez, J.A., Ramirez, N., Sola, J.J., et al. (2009). microRNA-451 regulates macrophage migration inhibitory factor production and proliferation of gastrointestinal cancer cells. Clin. Cancer Res. *15*, 2281–2290.

16. Sayed, D., Hong, C., Chen, I.Y., Lypowy, J., and Abdellatif, M. (2007). MicroRNAs play an essential role in the development of cardiac hypertrophy. Circ. Res. *100*, 416–424.

17. Zhao, Y., Samal, E., and Srivastava, D. (2005). Serum response factor regulates a muscle-specific microRNA that targets Hand2 during cardiogenesis. Nature *436*, 214–220.

18. Chen, J.-F., Murchison, E.P., Tang, R., Callis, T.E., Tatsuguchi, M., Deng, Z., Rojas, M., Hammond, S.M., Schneider, M.D., Selzman, C.H., et al. (2008). Targeted deletion of Dicer in the heart leads to dilated cardiomyopathy and heart failure. Proc. Natl. Acad. Sci. USA *105*, 2111–2116.

19. Rao, P.K., Toyama, Y., Chiang, H.R., Gupta, S., Bauer, M., Medvid, R., Reinhardt, F., Liao, R., Krieger, M., Jaenisch, R., et al. (2009). Loss of cardiac microRNA-mediated regulation leads to dilated cardiomyopathy and heart failure. Circ. Res. *105*, 585–594.

20. Zincarelli, C., Soltys, S., Rengo, G., Koch, W.J., and Rabinowitz, J.E. (2010). Comparative cardiac gene delivery of adeno-associated virus serotypes 1-9 reveals that AAV6 mediates the most efficient transduction in mouse heart. Clin. Transl. Sci. *3*, 81–89.

21. Chen, J.-F., Mandel, E.M., Thomson, J.M., Wu, Q., Callis, T.E., Hammond, S.M., Conlon, F.L., and Wang, D.-Z. (2006). The role of microRNA-1 and microRNA-133 in skeletal muscle proliferation and differentiation. Nat. Genet. *38*, 228–233.

22. Zhao, Y., Ransom, J.F., Li, A., Vedantham, V., von Drehle, M., Muth, A.N., Tsuchihashi, T., McManus, M.T., Schwartz, R.J., and Srivastava, D. (2007). Dysregulation of cardiogenesis, cardiac conduction, and cell cycle in mice lacking miRNA-1-2. Cell *129*, 303–317.

23. Lagos-Quintana, M., Rauhut, R., Yalcin, A., Meyer, J., Lendeckel, W., and Tuschl, T. (2002). Identification of tissue-specific microRNAs from mouse. Curr. Biol. *12*, 735–739.

24. Lee, R.C., and Ambros, V. (2001). An extensive class of small RNAs in Caenorhabditis elegans. Science *294*, 862–864.

25. Kuppusamy, K.T., Jones, D.C., Sperber, H., Madan, A., Fischer, K.A., Rodriguez, M.L., Pabon, L., Zhu, W.-Z., Tulloch, N.L., Yang, X., et al. (2015). Let-7 family of microRNA is required for maturation and adult-like metabolism in stem cell-derived cardiomyocytes. Proc. Natl. Acad. Sci. USA *112*, E2785–E2794.

26. Roberts, T.C., Blomberg, K.E.M., McClorey, G., El Andaloussi, S., Godfrey, C., Betts, C., Coursindel, T., Gait, M.J., Smith, C.I.E., and Wood, M.J.A. (2012). Expression analysis in multiple muscle groups and serum reveals complexity in the microRNA transcriptome of the mdx mouse with implications for therapy. Mol. Ther. Nucleic Acids *1*, e39.

27. Cheloufi, S., Dos Santos, C.O., Chong, M.M.W., and Hannon, G.J. (2010). A dicer-independent miRNA biogenesis pathway that requires Ago catalysis. Nature *465*, 584–589.

28. Cifuentes, D., Xue, H., Taylor, D.W., Patnode, H., Mishima, Y., Cheloufi, S., Ma, E., Mane, S., Hannon, G.J., Lawson, N.D., et al. (2010). A novel miRNA processing pathway independent of Dicer requires Argonaute2 catalytic activity. Science *328*, 1694–1698.

29. Liu, Y.P., Karg, M., Harwig, A., Herrera-Carrillo, E., Jongejan, A., van Kampen, A., and Berkhout, B. (2015). Mechanistic insights on the Dicer-independent AGO2-mediated processing of AgoshRNAs. RNA Biol. *12*, 92–100.

30. Börner, K., Niopek, D., Cotugno, G., Kaldenbach, M., Pankert, T., Willemsen, J., Zhang, X., Schürmann, N., Mockenhaupt, S., Serva, A., et al. (2013). Robust RNAi enhancement via human Argonaute-2 overexpression from plasmids, viral vectors and cell lines. Nucleic Acids Res. *41*, e199.

31. Giering, J.C., Grimm, D., Storm, T.A., and Kay, M.A. (2008). Expression of shRNA from a tissue-specific pol II promoter is an effective and safe RNAi therapeutic. Mol. Ther. *16*, 1630–1636.

32. Boudreau, R.L., Martins, I., and Davidson, B.L. (2009). Artificial microRNAs as siRNA shuttles: improved safety as compared to shRNAs in vitro and in vivo. Mol. Ther. *17*, 169–175.

33. Gabisonia, K., Prosdocimo, G., Aquaro, G.D., Carlucci, L., Zentilin, L., Secco, I., Ali, H., Braga, L., Gorgodze, N., Bernini, F., et al. (2019). MicroRNA therapy stimulates uncontrolled cardiac repair after myocardial infarction in pigs. Nature *569*, 418–422.

34. Harper, S.Q., and Davidson, B.L. (2005). Plasmid-based RNA interference: construction of small-hairpin RNA expression vectors. Methods Mol. Biol. *309*, 219–235.

35. Course, M.M., Gudsnuk, K., and Valdmanis, P.N. (2019). A Complete Pipeline for Isolating and Sequencing MicroRNAs, and Analyzing Them Using Open Source Tools. J. Vis. Exp. *150*, e59901.

36. Lewis, B.P., Burge, C.B., and Bartel, D.P. (2005). Conserved seed pairing, often flanked by adenosines, indicates that thousands of human genes are microRNA targets. Cell *120*, 15–20.

37. Langmead, B., Trapnell, C., Pop, M., and Salzberg, S.L. (2009). Ultrafast and memory-efficient alignment of short DNA sequences to the human genome. Genome Biol. *10*, R25.

38. Anders, S., and Huber, W. (2010). Differential expression analysis for sequence count data. Genome Biol. *11*, R106.

39. Livak, K.J., and Schmittgen, T.D. (2001). Analysis of relative gene expression data using real-time quantitative PCR and the 2(-Delta Delta C(T)) Method. Methods *25*, 402–408.

OMTN, Volume *19*

Supplemental Information

Endogenous MicroRNA Competition

as a Mechanism of shRNA-Induced Cardiotoxicity

Meredith M. Course, Kathryn Gudsnuk, Nitin Desai, Joel R. Chamberlain, and Paul N. Valdmanis

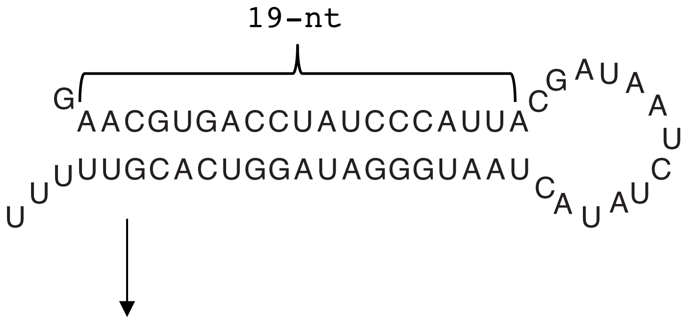

19-nt stem reads	Proportion of top reads
ATGGGATAGGTCACGTTTTTT	17.3%
ATGGGATAGGTCACGTTTTTTT	31.6%
AATGGGATAGGTCACGTTTTT	32.1%
AATGGGATAGGTCACGTTTTTT	14.4%

19-nt: GAACGTGACCTATCCCATTACG−−ATAATCTATACT−−AATGGGATAGGTCACGTTTTTT

21-nt: GAACGTGACCTATCCCATTACGTGATAATCTATACTGTAATGGGATAGGTCACGTTTTTT

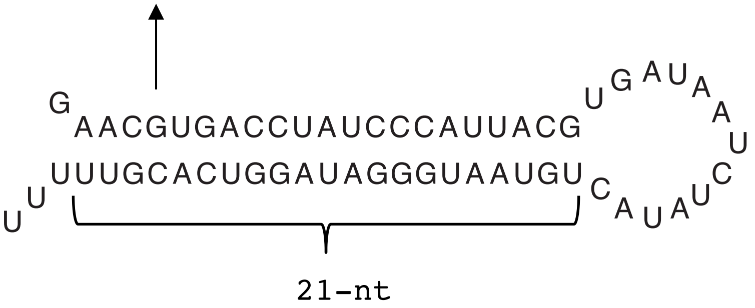

21-nt stem reads	Proportion of top reads
TAATGGGATAGGTCACGTTT	4.8%
TAATGGGATAGGTCACGTTTT	49.1%
TAATGGGATAGGTCACGTTTTT	33.7%
TAATGGGATAGGTCACGTTTTTT	7.6%

Figure S1. 19-nt and 21-nt sh-*lacZ* processing.
Shown are the predicted structures of the 19-nt and 21-nt hairpins, their flattened sequences, and the top four reads that are produced from the hairpins along with their relative abundance (n = 3, proportion is the mean average of reads in three hearts analyzed at 6-weeks post-injection). The reads produced from the 19-nt hairpin are not observed to an appreciable degree in the 21-nt-treated hearts, nor are the reads produced from the 21-nt hairpin observed in the 19-nt-treated hearts.

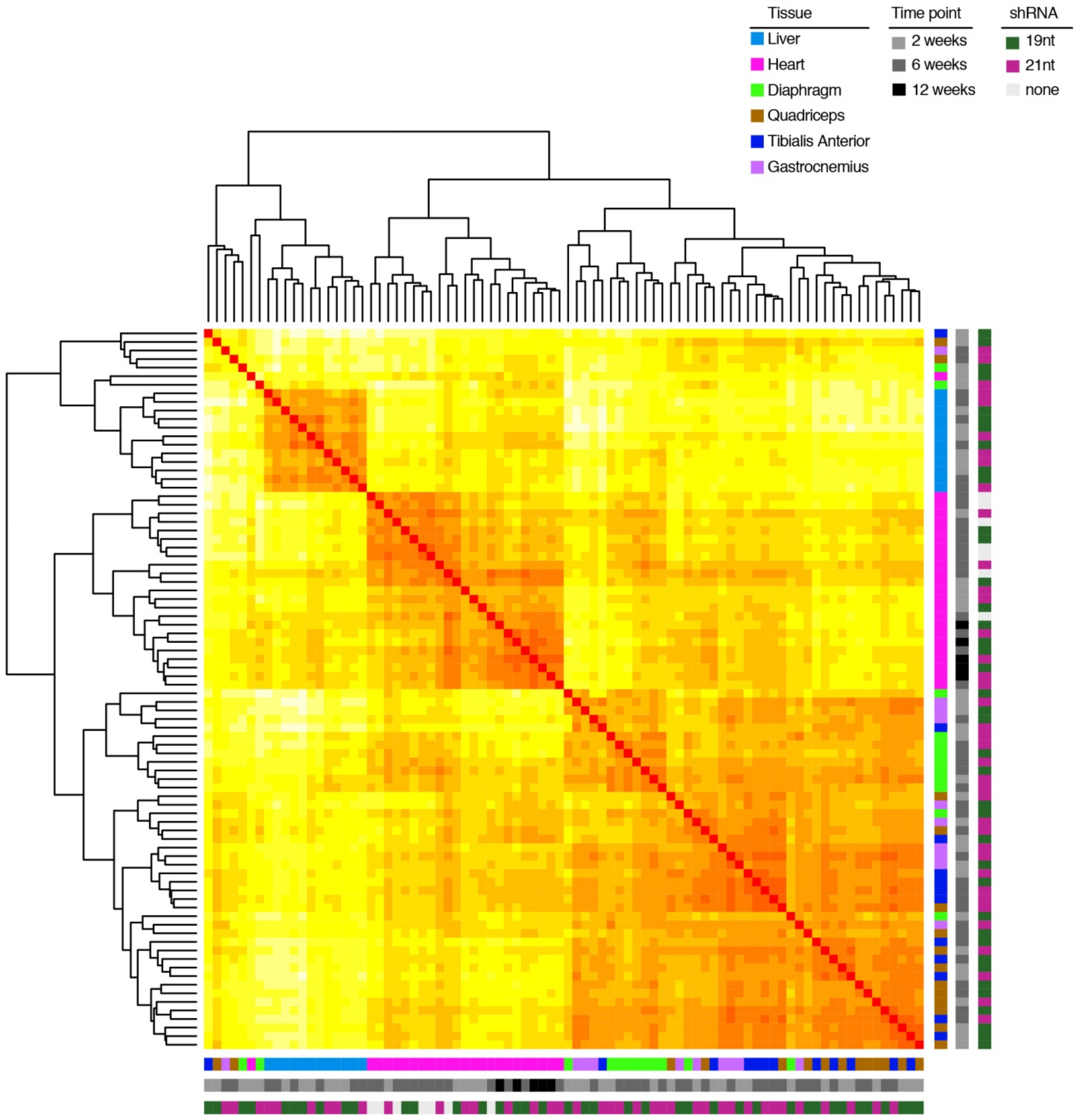

Figure S2. Hierarchical clustering of all samples. Tissue groups cluster into three broad categories: heart, liver, and all other muscles (diaphragm, quadriceps, tibialis anterior, and gastrocnemius). They do not cluster by time point (2, 6, or 12 weeks) or by treatment paradigm (no shRNA, 19-nt, or 21-nt).

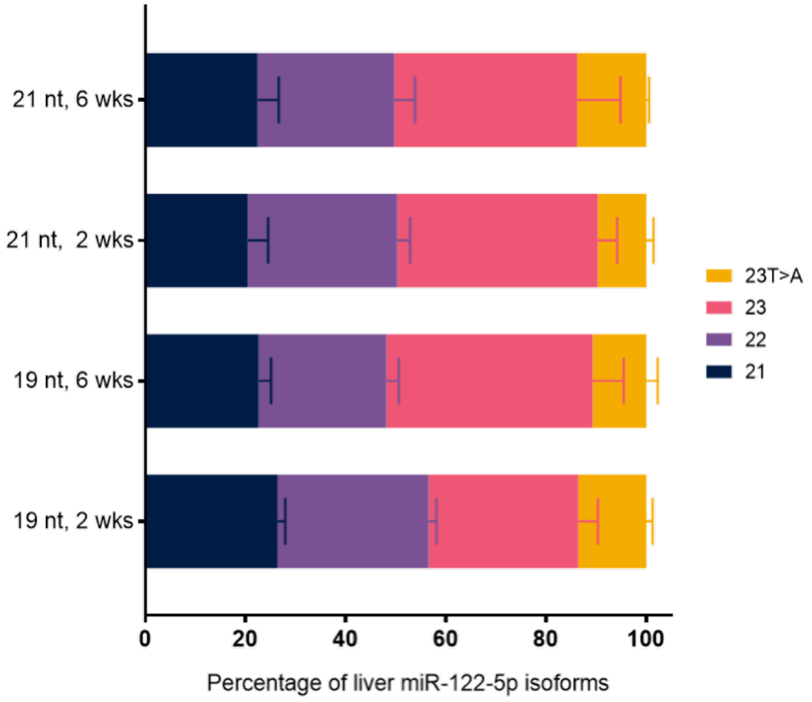

Figure S3. MiR-122-5p isoforms in liver tissues of 19-nt and 21-nt injected mice.
MiR-122-5p isoform expression in liver tissues after injection of the 19-nt shRNA and the 21-nt shRNA. The 22-nt isoform is not depleted relative to the 21-nt isoform. n = 3 mice per group. Data are mean ± SD.

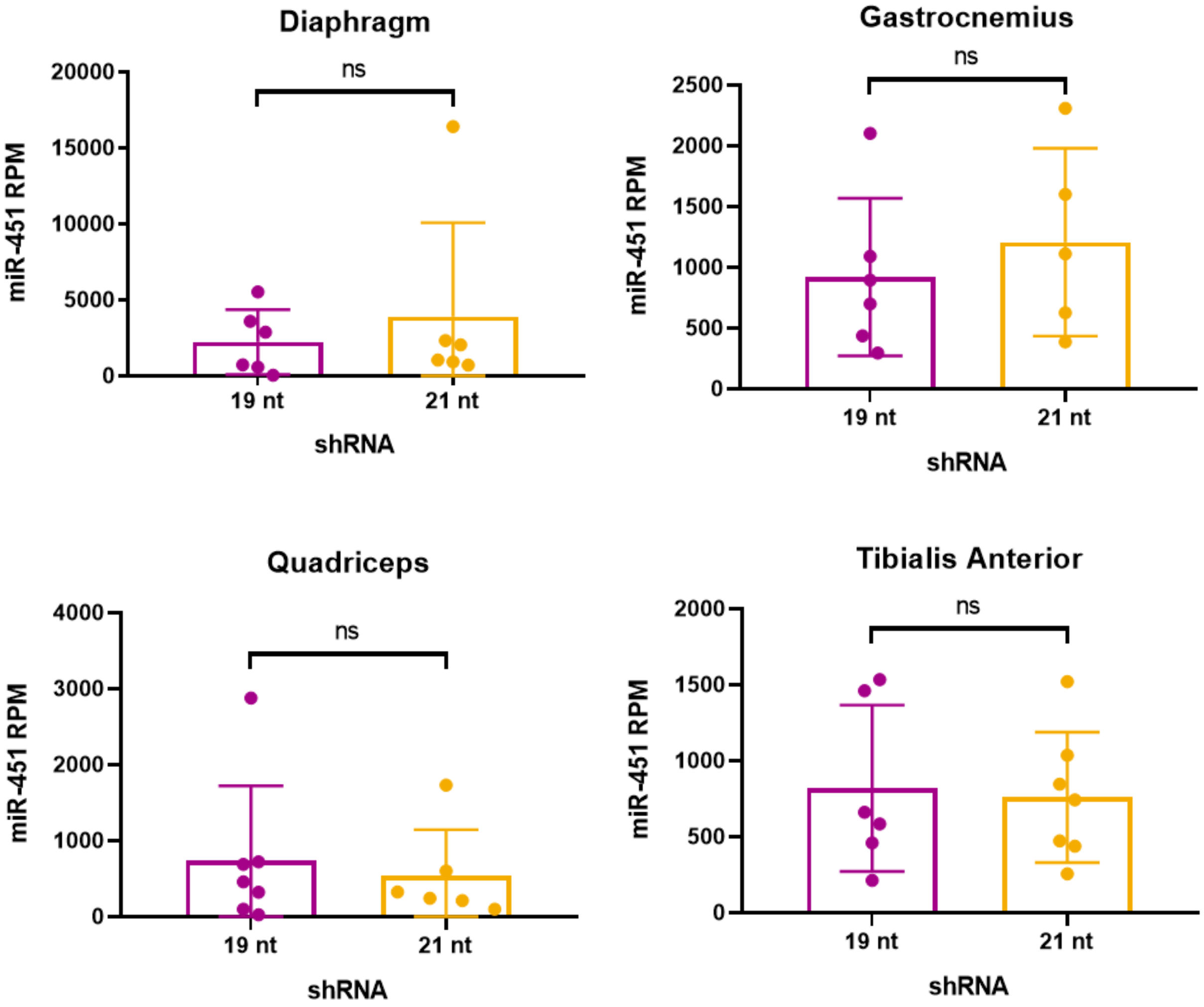

Figure S4. MiR-451 in muscle tissues of 19-nt and 21-nt injected mice.
MiR-451 expression in muscle tissues other than the heart after injection of the 19-nt shRNA and the 21-nt shRNA. n = 5-7 mice per group. Data are mean ± SD. RPM is reads per million. NS means “not significant,” as determined by a Student’s t-test.

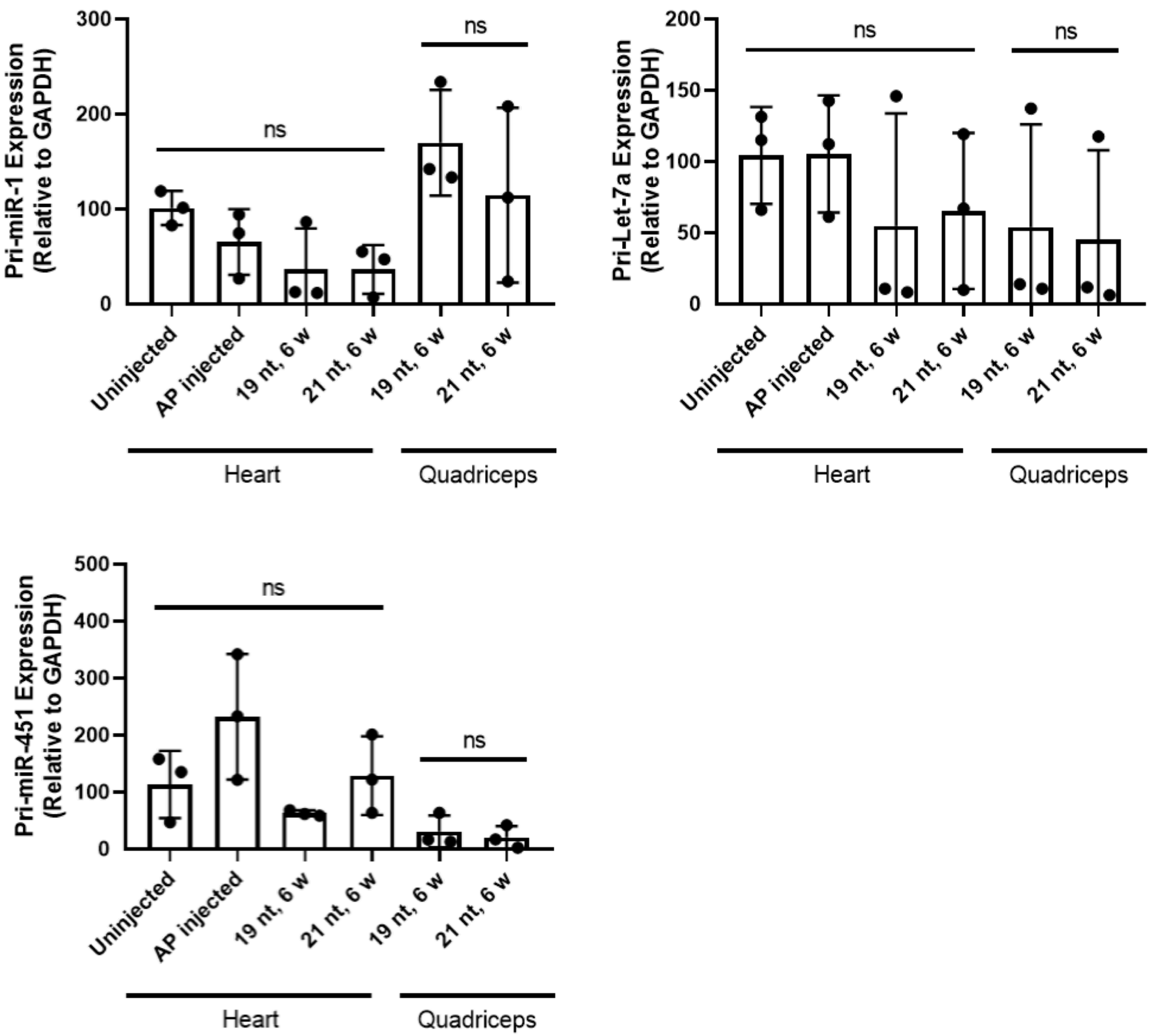

Figure S5. Primary miRNA transcripts in muscle tissues of 19-nt and 21-nt injected mice.
qRT-PCR for primary miRNA transcripts in heart and quadriceps tissues of 19- and 21-nt injected mice. n = 3 mice per group. NS means “not significant.” Analyses performed were one-way ANOVA tests followed by Tukey’s multiple comparisons for heart tissue, and Student’s t-tests for quadriceps tissue. Data are mean ± SD.
